# Addressing Challenges in *Chlamydia trachomatis* Detection: A Comparative Review of Diagnostic Methods

**DOI:** 10.3390/medicina60081236

**Published:** 2024-07-30

**Authors:** Rafaela Rodrigues, Ana Rita Silva, Carlos Sousa, Nuno Vale

**Affiliations:** 1 PerMed Research Group, Center for Health Technology and Services Research (CINTESIS), Rua Doutor Plácido da Costa, 4200-450 Porto, Portugal; rafaela24sofia@hotmail.com; 2 CINTESIS@RISE, Faculty of Medicine, University of Porto, Alameda Professor Hernâni Monteiro, 4200-319 Porto, Portugal; 3 Molecular Diagnostics Laboratory, Unilabs Portugal, Centro Empresarial Lionesa Porto, Rua Lionesa, 4465-671 Leça do Balio, Portugal; ana.rita.silva@unilabs.com (A.R.S.); carlos.sousa@unilabs.com (C.S.); 4 Department of Community Medicine, Health Information and Decision (MEDCIDS), Faculty of Medicine, University of Porto, Rua Doutor Plácido da Costa, 4200-450 Porto, Portugal

**Keywords:** *Chlamydia trachomatis*, sexually transmitted infections, molecular biology, screening, medico-diagnostic testing

## Abstract

Chlamydial infections are one of the most common sexually transmitted bacterial infections worldwide, which is related to serious consequences for the mental, sexual, and reproductive health of women and men. The infection is commonly asymptomatic; consequently, screening programs for infection control have been introduced in some countries. The detection methods of *Chlamydia trachomatis* infections have evolved since the establishment of the first gold-standard detection method in the 1970s, the culture assay. Over the decades, many efforts were made to find methods with a higher sensitivity, until the 1990s, when, as a result of advances in molecular biology, nucleic acid amplification tests came into use with more sensitivity, and, currently, there are several available with which to detect infection. Therefore, herein, we will review the main methods used for CT detection and the differences between them, in terms of targets, infections that can be detected, sensitivity, and specificity. We will focus on some of the FDA-approved CT detection tests and highlight the major advantages and superiority of using molecular biology techniques. In addition, we will examine the larger challenges and limitations of the methods currently in use and discuss how they might be surpassed. Moreover, in this review, we will describe the next step to carry out after testing positive for CT infection.

## 1. Introduction

**Figure 1:**
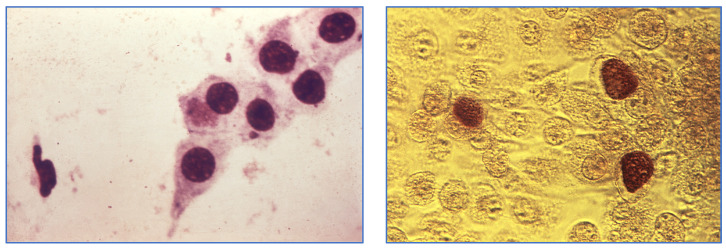
**Left**: This photomicrograph of *Chlamydia trachomatis* is taken from a urethral scrape. Note the presence of a cluster of spore-like C. trachomatis elementary bodies located intracellularly, inside one of the larger epithelial cells. Source: CDC/Dr. Wiesner, Dr. Kaufman. **Right**: Under a magnification of 200×, this photomicrograph depicts a view of a McCoy cell mono-layer culture, which had been inoculated with *Chlamydia trachomatis* bacteria, and subsequently developed these intra-cellular *C. trachomatis* inclusion bodies. Source: CDC/Dr. E. Arum; Dr. N. Jacobs. These images are in the public domain and, thus, free of any copyright restrictions.

Among the most common sexually transmitted bacterial infections globally, *Chlamydia trachomatis* (CT) is the causative agent of a variety of pathogeneses (Figure 1), the most common of which occur in the genital and urinary tract, such as cervicitis, urethritis, pelvic inflammatory disease, or proctitis, but it can also occur in the throat and rectum. Another occurrence, in the eyes, is trachoma (chronic conjunctivitis, which is a main cause of blindness), and, in newborns, it can cause pneumonia and/or conjunctivitis [1,2,3]. Interestingly, recently, Gallenga and colleagues have published a brief historical review of the extra-genital manifestations of CT infections, especially in the eyes. The authors stated that, historically, trachoma was primarily attributed to transmission by flies, such as *Musca sorbens* and *Scatophaga stercoraria*; therefore, the World Health Organization (WHO) has developed the SAFE strategy (Surgery, Antibiotics, Facial cleanliness, and Environmental improvement) to combat this ophthalmological disease. Notwithstanding, over the years, new evidence has been gathered indicating that CT also plays a significant role in conditions such as sterile chronic prostatitis, conjunctivitis, oropharyngitis, and proctitis. In line with these findings, the WHO has added to the SAFE strategy with one designated SAFE-S (Surgery, Antibiotics, Facial cleanliness, Environmental improvement, and Sexual behavior), where the relevance of addressing sexual transmission routes of the infection is emphasized, especially in asymptomatic individuals with high-risk sexual behaviors, in order to enhance awareness and the prevention of sexually transmitted infections [4].

In addition, CT is responsible for a specific ulcerative disease of the genital area, known as lymphogranuloma venereum (LGV), which is characterized by severe proctocolitis or enlarged lymph nodes, and is less common and caused, in particular, by LGV strains (L1 to L3) [3,5]. Untreated infections can lead to severe and irreversible outcomes, such as, in women, tubal infertility, chronic pelvic pain, ectopic pregnancies, and pelvic inflammatory disease, which result in high medical costs and place a psychological burden on patients [6,7].

It is noteworthy that diagnosis rates for chlamydial infections continue to rise in the developed world, which could be explained, in part, by the asymptomatic nature of the infection. Especially, women are asymptomatic in more than 75% of cases for the condition, while, in men, chlamydial infection is usually symptomatic in 50% of cases, although it can cause prostatitis and chronic pain, and increase the risk of acquiring and transmitting HIV [8,9,10]. Accordingly, some countries have implemented CT control measures at several levels. The first is primary prevention, which includes warning about risks, promoting condom use, and sexual health habits. The next level is case management, where the appropriate prevention, diagnostic, clinical, and partner notification services are provided. And, finally, there are screening programs, in a specific target group, to identify and treat those infected in time to avoid transmission and severe complications. Nonetheless, the late strategy came with some challenges and limitations that are still under debate [11]. First of all, these screening programs mostly focus on urogenital infections, which could lead to extra-urogenital infections going undetected. In line with this, some authors refer to the importance of the adaptation of different types of strategies to carry out the screening, especially, depending on sexual behaviors or sexual orientation [12]. In addition, some authors reported that the strategy of testing and treating might not be as appropriate because cases that can sometimes be resolved spontaneously are overtreated [13,14,15,16].

The most effective strategy for combating infections is through vaccination, which provides individual immunity to the population and is crucial for controlling and eradicating pathogens that pose public health threats. Despite the dedicated efforts of researchers, an effective and safe vaccine against CT has not yet been developed [17,18]. However, there is ongoing research into the in silico design of a novel multi-epitope vaccine candidate. Preliminary results from immune simulations are promising, offering hope for CT control, but further evaluation through in vitro and in vivo studies is needed to verify these findings and pave the way for potential CT eradication [19]. Interestingly, phase 1 clinical trials have demonstrated the safety and tolerability of a chlamydia vaccine candidate CTH522; therefore, phase 2 studies must be pursued [20].

In this article, we describe and compare CT laboratory detection methods, used for screening and diagnosis, with a particular emphasis on standard methods using FDA-approved nucleic acid amplification technology, and highlight the major advantages and superiority of these molecular biology techniques. In addition, we scrutinize the larger challenges and limitations of the methods currently in use and discuss how these might be overcome. With this review, we not only contribute to show the evolution of CT detection methods over the last decades, but also summarize the main features of some FDA-approved tests for the laboratory detection of this infection, evidencing the main challenges and limitations of these available tests, which are very important for the diagnosis and screening of chlamydial infection.

## 2. Detection Methods

CT is an obligate, intracellular, Gram-negative bacterium, whose serovars recognized are 19, based on the specific epitopes encoded by the ompA gene regarding the major outer membrane protein (MOMP): serovars A to C cause trachoma; D to K are associated with urogenital, ocular, and rectal infections; and L1 to L3 are associated with LGV [21,22].

CT infection is often asymptomatic, meaning that most cases are detected through screening. Screening and diagnostic methods for chlamydial infection began in the 1970s using the culture method, which was considered the gold standard at that time [23]. However, due to the lack of sensitivity shown by false-negative tests and the complexity of the assay, researchers began developing alternative methods. Specifically, direct fluorescence and enzyme immunoassays were first explored [24]. One of the disadvantages of these newly developed approaches is its lower sensitivity, compared with cell culture. Later, in the 1990s, during the era of molecular biology advances, molecular approaches such as polymerase chain reaction (PCR) emerged, bringing about improved accuracy up to the present day [25]. Currently, not all of these methods could be pursued to detect CT, although we will further describe in more detail the most common ones used in the past vs. the present.

### 2.1. Cell Culture Methodology

**Figure 2:**
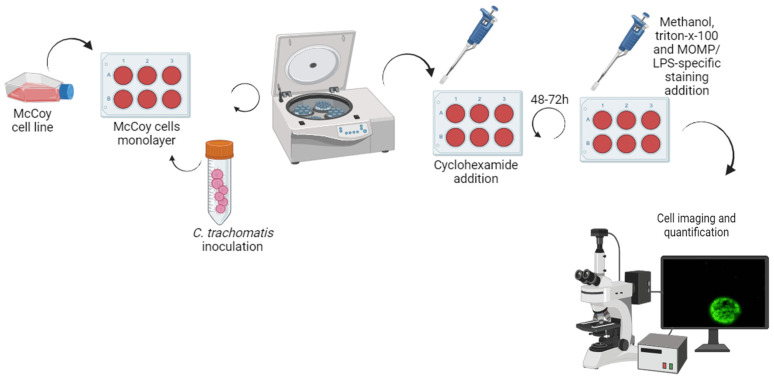
Summary of the key steps of McCoy cell culture method for CT detection by fluorescent antibody staining. Briefly, when the McCoy cells form a monolayer in the wells, the patient sample is added. Then, the plate is centrifuged for 4–5 h to enhance infection of the cell monolayer. After, cycloheximide is added to stop host cell division for 48–72 h. Finally, the cells are fixed with methanol, disrupted with triton-x-100, and stained with specific fluorescein-conjugated monoclonal antibodies (anti-MOMP or anti-LPS) to allowing specific visualization of CT inclusions through fluorescent microscopy. Figure created using BioRender, version 04 [31].

The first used method for chlamydial infection detection was first performed using the yolk-sac inoculation of embryonated eggs, that was later replaced with cell culture, whose protocol could vary according to the laboratory. It is difficult to standardize; however, it is generally based on the inoculation of a volume of a suitable specimen, such as an endocervix, urethra, anal canal, or conjunctivae swab, with an established monolayer of McCoy cells (HeLa 229 or Buffalo Green Monkey Kidney cells), treated with cycloheximide [26]. Of note, the McCoy cell culture is the most used method (Figure 2). After 48 to 72 h of incubation, a dark-ground microscopy technique was used after fluorescence-labelled antibodies to chlamydial antigens (Giemsa staining or iodine are not recommended by their lower specificity), allowing us to search for the intracytoplasmic inclusion bodies [23,27]. This method has several challenges and limitations; not only is it dependent on laboratory conditions, but it is also dependent on the sample collection and preservation, a rapid cold transport system is required, and the procedure is demanding and time-consuming, increasing the test turnaround time, and it also requires a trained microscopist. The test sensitivity varies between 50 to 70% and it is relatively expensive [28]. Therefore, currently, this method is only performed for research [23,29]. Following the CDC guidelines for *Chlamydia trachomatis* detection, in 2014, a protocol is recommended which included the following steps [30]: (i) specimen collection swabs for the CT culture must have a plastic or wire shaft and either a rayon, dacron, or cytobrush tip, in order to not inhibit isolation; (ii) specimen collection for CT culture is invasive—for example, the insertion of a swab of 2–3 cm into the male urethral or 1–2 cm into the endocervical canal followed by two or three rotations; (iii) following the sample collection, it should be stored in an appropriate transport media such as sucrose phosphate glutamate buffer or M4 media (Thermal Scientific, Lenexa, KS, USA) and transported at ≤4 °C to the laboratory within 24 h of collection or stored at −70 °C if transport is delayed >24 h; (iv) the specimen is inoculated by centrifugation onto a confluent monolayer of McCoy, HeLa 229, or Buffalo green monkey kidney cells; (v) once the specimen has been inoculated, 2 µg/mL of cycloheximide should be added to the growth medium; (vi) after 48–72 h of growth, if staining is planned, cells inoculated with the specimen can be directly visualized without the need for harvesting—successful infections typically result in the development of intracytoplasmic inclusions containing a substantial number of CT elementary and reticulate bodies; (vii) after the growth period, cell monolayers need to be fixed and disrupted to allow the antibody recognition of both chlamydial and host cells—fluorescein-conjugated monoclonal antibodies, either genus-specific or species-specific (anti-MOMP or anti-LPS), are then applied to enable the specific visualization of chlamydial inclusions using an epifluorescent microscope; and, finally, (viii) the staining of the cellular nucleus is carried out using specific antibodies (DAPI).

### 2.2. Direct Fluorescent Antibody

**Figure 3:**
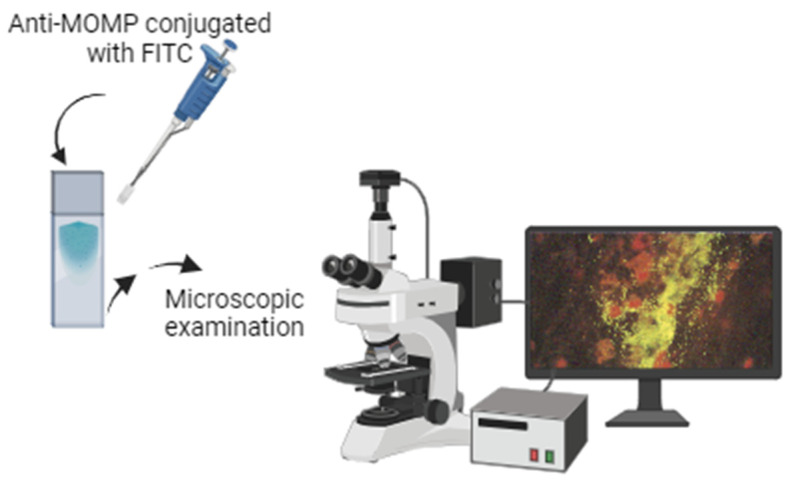
Key steps of DFA technique to CT detection. Starting from a cervical specimen from a patient with CT infection, the swab was smeared [34]. For the staining, the samples are incubated with anti-MOMP-FITC. The slide was examined using fluorescent microscopy, searching for elementary bodies (the points that mark the fluorescent color and that represent infectious structures are looked for). Figure created using BioRender.

In the era of monoclonal antibody techniques, non-culture methods for CT detection begin to arise. One of these techniques is the direct fluorescent antibody (DFA) technique, associated with a lower cost, the simplest process, and easier sample transport (it does not require cold transportation). Notwithstanding the fact that there are several disadvantages associated with the subjectivity of the examination, the artifacts that could be present in the slide may influence the test result, delays in the process, and the suboptimal diagnostic accuracy [32,33]. DFA is based on the direct detection of CT cellular smears using specific conjugated antibody-fluorescent molecules (Figure 3), whose target site could be MOMP (Syva Microtrak, Rancho Cordova, CA, USA; Trinity Biotech, Bray, Ireland) or lipopolysaccharides (LPS) (Kallestad, Waupaca, WI, USA) molecules, depending on the commercial kit used [32]. In brief, the main steps of the DFA protocol are represented in Figure 3. In detail, the main steps of the protocol are as follows [30]: (i) the obtention of samples that could be from different anatomical sites, preferably an endocervical specimen (women) or a urethral specimen (men); (ii) the swabs were smeared on a glass slide that was then dried and fixed with methanol for 10 min; (iii) the slides can be stored at RT; and, (iv) within 7 days, the staining must be processed. For the staining, the slides were first treated with a detergent solution (Triton X-100 or Tween 20) to disrupt the cells and enhance antibody recognition. After the detergent treatment, the slides were incubated with the fluorescent-labeled monoclonal antibody (anti-MOMP-FITC) for 30 min at RT; (v) then, it was washed in distilled water and mounted following the recommendations; and (vi) the slides were then examined by fluorescent microscopy for elementary bodies (EBs), represented in green color.

The test sensitivity and specificity vary. Specifically, the labeled antibodies must bind to the CT elementary bodies, but anti-LPS monoclonal antibodies might cross-react with other bacteria, causing false-positive results to arise, associated with a lower specificity [30]. Notwithstanding, this is operator-dependent, and, with an experienced microscopist, the diagnostic accuracy could increase [35]. DFA should not be used for the routine testing of CT infections [30].

### 2.3. Enzyme Immunoassay

**Figure 4:**
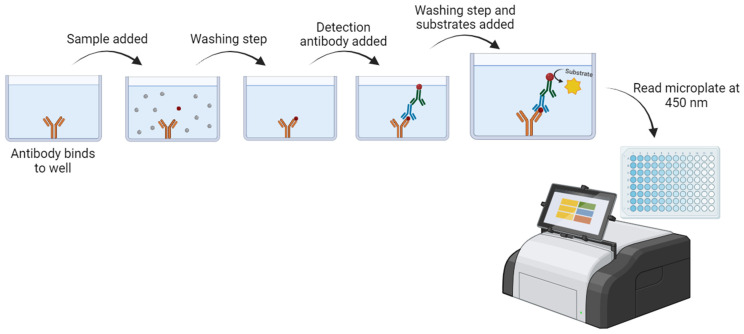
Enzyme immunoassay (Micro Trak enzyme immunoassay) for CT detection. First, the plates should be coated with the capture antibody; then, the sample is added, and, if the antigen is present, the binding process occurs. Finally, the detection antibody is added, and binds to the antigen (if present). The last step is the substrate addition, which reacts with the enzyme conjugate, producing a product that can be measured (reading the microplate at 450 nm) [42]. Figure created using BioRender.

Another non-culture method first developed was enzyme immunoassays (EIAs), using commercial immunoassays for the direct detection of CT, which is based on the CT antigen detection in urethral or cervical swabs, using commercially available kits [36]. It was developed for the rapid (automated method), large-scale screening, cost-effective, and simple diagnosis of chlamydial genital infection [37]. However, it has a suboptimal diagnostic accuracy, with a sensitivity of around 65%, and it is associated with higher costs [24]. The first developed method was Chlamydiazyme (Abbott Laboratories, Hong Kong, China), using polyclonal antibodies, which was later found to have a cross-reactivity with other pathogens naturally found in vaginal and rectal flora [38,39,40]. Moreover, some monoclonal antibody techniques were developed, such as Micro Trak, a test based on the use of a chromophore-conjugated monoclonal antibody against LPS of CT [41]. The procedure is simple and dependent on the manufacturer’s instructions. Briefly, as illustrated in Figure 4, these are the key steps [41]: (i) carry out the resuspension of the sample in a washing buffer, digestion, and treatment with a solution for elution and solubilization; (ii) the plates should be coated with the capture antibody (anti-LPS) to be incubated with the previous sample solution prepared; (iii) incubate the samples in the coated plates at 37 °C (the ligation step could occur between the antibodies and the antigens, if present), and then wash the plates; (iv) carry out an incubation step with an enzyme-conjugated antibody (peroxidase-labeled anti-IgG), followed by a washing step; (v) carry out the addition of enzyme substrates (TMB and peroxide), followed by a washing step; and (vi) stop the reagent addition, and measure the sample absorbance at 450 nm.

Importantly, for the above-mentioned reasons, in detail, along with the high probability of false-positive results, concomitantly with the lower test sensitivity (65–75%), all of these enzyme immunoassays are not currently in use. 

### 2.4. Serology

Serology tests, in the past, were useful for lymphogranuloma venereum (LGV) diagnosis [43]—specifically, the micro-immunofluorescence (MIF) assay, that is an indirect fluorescent antibody technique, although this is not currently used due to the lower specificity [42]. Additionally, the complement fixation test (CFT) is a serologic method for the diagnosis of LGV and psittacosis, a respiratory infection caused by the bacterium *Chlamydia psittaci*. The CFT measures the Chlamydia genus antibody. Briefly, the test is based on the patient serum mix with a specific antigen, and a complement is added. After this, a binding step will occur between the antigen and the antibody (if present in the serum), resulting in immune complexes. Subsequently, the complement system is activated and binds to the immune complexes. Finally, a non-binding complement is measured by adding erythrocyte cells, and the test result is based on the hemolysis level [44,45].

### 2.5. Nucleic Acid Hybridization

There are several commercially available nucleic acid hybridization tests for CT detection that rely on the hybridization between oligonucleotide sequences specifically designed to bind to complementary sequences in the target CT DNA. Typically, a nucleic acid probe-based chemiluminescent assay is employed, and, upon successful hybridization, the fluorescent signal is amplified. For example, Molano and colleagues have developed a nucleic acid hybridization assay in a reverse line blot (RLB) assay for the detection and typing of 14 CT genovars. In detail, they have used a colorimetric multiplex PCR-RLB assay specifically for the VD2 region of ompA, allowing for the identification of 14 CT serovars [46].

A commercially available test following this methodology is the Digene HC2 CT/GC DNA Test (Qiagen), that enables the detection of *Chlamydia trachomatis* and *Neisseria gonorrhoeae* (NG). Briefly, the main steps of this specific test are as follows [47]: (i) the hybridization between the sample target DNA and CT/NG RNA probes; (ii) the capture of RNA–DNA hybrid complexes on a coated microplate; (iii) the reaction with alkaline phosphatase-conjugated antibodies specific for RNA–DNA hybrids; (iv) the detection with a chemiluminescent substrate; and (v) the emitted light being measured using a luminometer.

Notwithstanding, these approaches have several limitations, such as the contamination risk, the expenses, the need to standardize reagents, and the physical requirements of the laboratory. It is only used as a research tool and not for diagnostic purposes [25,33].

### 2.6. Nucleic Acid Amplification Test

Currently, the gold standard for screening and diagnostics are nucleic acid amplification tests, including DNA amplification tests (through polymerase chain reaction—PCR or strand displacement amplification—SDA) and RNA amplification tests (through transcription-mediated amplification—TMA), associated with a high sensitivity [3,48,49]. For the PCR method, the target DNA sequences used for the diagnosis are located between the 16S and 23S rRNA genes, and also in the *omp1* and cryptic plasmid. In the first phase, the CT DNA extraction from the samples to be amplified occurs, performed according to the manufacturer’s instructions using commercially available kits for nucleic acid extraction and purification. Briefly, for urine samples, this process starts with the centrifugation of the samples; then, the supernatant is discarded, and proteinase K is added for 10 min. DNA should be immediately automatically purified using supermagnetic bead techniques and amplified by PCR (as described below). The amplified DNA targets are detected through a colorimetric evaluation, using probes labeled with different fluorophores, which hybridize with the complementary sequence of the target, resulting in a fluorescence emittance (positive result). In addition, as presented in Table 1, the Aptima assay (Hologic) is a transcription-mediated amplification (TMA) method, one of the most widely used strategies for the CT diagnosis. In brief, the TMA technique is used for the isothermal amplification of RNA. Briefly, the process begins with the action of a reverse transcriptase enzyme, enabling RNA to undergo complementary DNA (cDNA) conversion. Then, the RNA polymerase enzyme synthesizes multiple RNA amplicons through an isothermal amplification that is finally detected by fluorescent probes or molecular beacons [50]. These tests have a higher sensitivity, specificity, and accuracy than the above-mentioned methods. However, these technologies have some disadvantages associated: they take more time, they are more expensive, and they require trained technicians and complicated laboratory equipment [8]. Therefore, they are not available for all communities, namely, in developing countries, due to the higher associated costs and expertise required [51,52].

**Figure 5:**
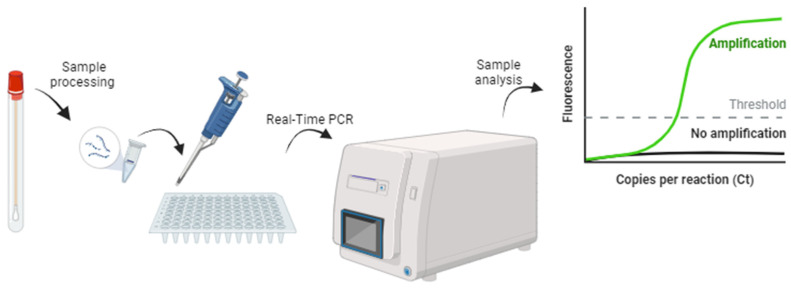
NAAT key steps to CT detection. Briefly, the process starts with DNA extraction followed by Real-Time PCR in order to obtain the Ct values to analyze the presence of CT [3]. Figure created using BioRender.

The majority of NAATs use a Real-Time PCR technique that is based on the following steps, also illustrated in Figure 5: (i) the sample collection and preparation for DNA extraction using commercially available kits; (ii) the preparation of a master mix with TaqMan probes, Taq DNA Polymerase, primers, dNTPs, and PCR buffer, following the manufacturer’s instructions; (iii) the addition of the master mix to the samples, as well as the controls, if provided by the test kit; (iv) PCR amplification for the CT nucleic acid replication and monitoring fluorescence; and (v) the determination of the cycle threshold (Ct) values for each sample to determine if the sample is positive or negative for CT.

There are several commercially available NAATs with distinct characteristics; therefore, some of those, FDA-approved, are described and compared in Table 1. It is worth noting that the test chosen should allow for a dual test, *Chlamydia trachomatis* and *Neisseria gonorrhea* detection, because co-infection could occur.

To the best of our knowledge, within those that are FDA-approved, the major NAATs for CT detection that are commercially available are Abbott RealTime, Becton Dickinson ProbeTec, GenProbe Aptima Combo 2, and Roche Diagnostics Cobas [53].

**Table 1:** FDA-approved NAATs commercially available for detecting CT and other STIs [54,55,56,57,58,59,60,61,62,63].

NAAT	Target Sequence	Infections Detected	Sample Type	Sensitivity	Limitations	Control	Method
Becton Dickinson MAX CT/NG/TV (Franklin Lakes, NJ, USA) [54,55]	2 CT and 2 NG gene targets; 1 TV gene target	CT NG TV	Vaginal, endocervical, and gynecological swab; female urine specimens	>98.7%	Less sensitivity using urine samples; co-infection might impact test performance	Sample processing control	Real-Time PCR
Alinity m STI Assay (Abbott Molecular, Inc.; Des Plaines, IL, USA) [56]	CT rRNA; NG gDNA; rRNA TV; rRNA MG	CT NG TV MG	Vaginal, endocervical swabs; male urine	100%	False-negative test could occur for MG, when the sample is an endocervical swab	Exogenous internal and cellular controls; positive and negative control	Real-Time PCR
Abbott RealTime CT/NG (Abbott Molecular Inc.; Des Plaines, IL, USA) [57]	CT plasmid DNA; NG gDNA	CT NG	Vaginal swab; urine specimens	95.2–97.5%	Endocervical and female urine specimens are associated with lower sensitivity	Internal control; negative control; cutoff control	Real-Time PCR
COBAS CT/NG (Roche Molecular Systems, Inc.; Rotkreuz, Switzerland) [58,59,60]	CT DNA (cryptic plasmid plus *ompA*); 2 sequences in DR9 region of NG DNA	CT/NG	Urine, pharyngeal, rectal, endocervical, and vaginal samples	95.6–100%	Relatively low oropharyngeal loads of NG could not be detected	Internal control; AmpErase enzyme—contamination control	Real-Time PCR
APTIMA Combo 2 Assay (Hologic Gen-Probe, Inc.; Marlborough, MA, USA) [61]	CT 23S rRNA and NG 16S rRNA	CT/NG	Endocervical, vaginal, throat, rectal, and male urethral swab; urine specimens	94.2–99.2%	Still requires a laboratory-based platform (Panther System)	Positive and negative control for CT/NG	Transcription-Mediated Amplification
BD ProbeTec ET CT/NG Amplified DNA Assays (Becton Dickinson; Franklin Lakes, NJ, USA) [62]	CT cryptic plasmid DNA and NG pilin gene DNA sequence	CT/NG	Endocervical and urine specimens	90.7–96%	Only allow genital infection diagnosis	Amplification control	Strand Displacement Amplification
Xpert CT/NG (Cepheid; Sunnyvale, CA, USA) [63]	CT DNA and 2 targets of NG DNA	CT/NG	Vaginal, endocervical, oropharyngeal, and rectal swabs; urine samples	95.6–100%	It is not currently a CLIA-waived test (it must be performed in specific laboratories used to moderate- or high-complexity testing)	Sample processing control, sample adequacy control, and probe check control	Real-Time PCR

MG—Mycoplasma genitalium; TV—Trichomonas vaginalis; NG—Neisseria gonorrheae; CT—Chlamydia trachomatis.

### 2.7. Near-Patient Testing

Currently, CT infection screening is particularly recommended for women younger than 25 years of age who were sexually active, and women at risk (specific groups in the population who have a risky sexual behavior) [64]. To be better accepted and effective, the screening should be cost-effective, accurate, and rapid, providing results within minutes; in a clinic, this could be achieved using near-patient tests and point-of-care tests (POCT) [65]. At present, the available near-patient test is the GeneXpert *Chlamydia trachomatis*/*Neisseria gonorrhoeae* Platform; despite the necessity of this platform to be installed on a laboratory usually, it could be installed in a point of care, such as a hospital, making this test a better option due to its relatively rapid turnaround time (approximately 45–90 min) [66,67]. Moreover, some authors report that this test is the one that provides the higher sensitivity (>98.7%) due to the nucleic acid amplification method it uses [68]. It is widely used for rectal and pharyngeal specimens, because it is the only FDA-approved one for these sample types. The disadvantage of this test is the fact that it is not a POCT, and, currently, it does not have a CLIA-waived status, meaning that, in fact, it cannot be used out of a clinical laboratory [69]. Of note, the CLIA-waiver requirements refer to regulations established by the Clinical Laboratory Improvement Amendments (CLIA) of 1988 in the United States, and its importance is because they govern the certification and oversight of clinical laboratories as those that perform diagnostic tests [70]. A POCT currently used is the Binx io Platform, a CLIA-waived test by the FDA; thus, it can be performed near the patient in a healthcare unit. This test is based on the isothermal nucleic acid amplification technology (LAMP or loop-mediated isothermal amplification) that takes place at a single, constant temperature, simplifying the process and reducing the need for complex equipment, for the rapid and accurate detection of the pathogens. Its sensitivity is around 96.1% and the turnaround time is approximately 30 min [71]. Later, a Visby Medical sexual health test (Visby Medical, San Jose, CA, USA) has been developed, cleared by the FDA, to detect CT, NG, and TV [68,69]. This test brings new advantages because it accomplishes all the requirements of a POCT described by the WHO: it is rapid (around 30 min) and robust, equipment-free, sensitive (97.4–99.2%), and specific (96.9–99.4%) [65,68,72]. Notwithstanding, it is only approved for vaginal swabs, and its performance for NG detection was suboptimal (sensitivity around 66%) [73].

The disadvantage of these approaches could be that some of them have a lower sensitivity compared to NAATs. Nevertheless, there are simulation studies that show that, despite the lower sensitivity, these approaches are more cost-effective and are associated with higher treatment success that reduces the loss of follow-up, as the turnaround time provides the result in time to treat the patient appropriately when needed at a patient appointment. And it has been proven that timely diagnoses can be the key to effective treatment. In addition, this is also important because it breaks the chain of transmission and could avoid complicated sequelae [74,75,76].

### 2.8. Next Step after Testing Positive for Chlamydia trachomatis

*Chlamydia trachomatis* is the most significant cause of bacterial sexually transmitted infections all over the world. In addition, due to its asymptomatic nature, many cases remain undetected, which leads to not only the underestimated prevalence, but also undertreated cases [77]. The diagnostic methods are essential in order to have an early detection of the infection, allowing CT treatment for infection control. The treatment is simple and usually effective in most cases. If there are complications, a different and longer treatment is required. As we stated in a previous work, following CDC guidelines for CT treatment, the first-line treatment is doxycycline, with the exception of pregnant women and neonates’ infections [18,78]. This drug is contraindicated during pregnancy; thus, azithromycin must be prescribed in those cases. Moreover, when the adhesion to the treatment is a problem for the patient, the latest must be considered due to the single-dose regimen. For neonates, erythromycin or ethylsuccinate are the recommended drugs [79]. To detect therapeutic failure, follow-up is recommended but only in some specific cases, such as in pregnant women and infants. Importantly, following the guidelines, the treatment regimen recommended is based on a single drug for an uncomplicated infection. Notwithstanding, in the cases where the infection has evolved to PID, a combination of drugs is recommended, specifically, one of the following schemes: ceftriaxone plus doxycycline plus metronidazole, or cefotetan plus doxycycline, or cefoxitin plus doxycycline [78]. Moreover, all the patients that test positive for chlamydial infection must abstain from sexual activity for 7 days after treatment and their partners must be also tested and treated if needed [79].

## 3. Discussion

Sexually transmitted *Chlamydia trachomatis* infection is currently a vast problem worldwide, associated with high health costs and serious sequelae if not treated. This results in the need to detect and treat this infection in time. Gallenga et al. provided a comprehensive historical review highlighting the evolution of understanding CT transmission routes, and the implementation of the WHO’s SAFE strategy, which emphasizes Surgery, Antibiotics, Facial cleanliness, and Environmental improvement. Such a strategy has been pivotal in trachoma management, traditionally associated with eye-seeking flies such as *Musca sorbens* and *Scatophaga stercoraria* [4,80]. Importantly, the current persistence of extra-genital CT infections in high-income countries underscores the ongoing challenges in this field, as defended by Garcia-Teillard et al. [81]—in detail, the need for targeted antibiotic therapies to combat emerging resistance patterns and the environmental impact of pesticide use, which affects biodiversity and human health [82]. Additionally, the authors also argue that the new WHO strategy, SAFE-S, is essential due to the change in sexual behaviors that is observed currently [4,81,82].

Historically, since the 1990s, CT infection has been the most frequently reported STI in the USA and Europe [24]. At that time, the diagnosis was carried out using the cell culture method, which had a lower sensitivity, despite their higher specificity. Hence, there was an urgent need to develop new methods with a higher sensitivity to reduce the number of false-negative results, thus breaking the chain of transmission and treating infected individuals appropriately to avoid severe sequelae [23]. In fact, several alternative methods of CT detection, based on antibodies and immunoassays, have been developed, but none of them has the required sensitivity [24,35]. Later, in the era of molecular biology, the highest sensitivity was achieved with techniques based on DNA technologies, namely, NAATs [83]. Nevertheless, this approach has some challenges that need to be addressed, related to the turnaround time of the results, as these delays affect patient follow-up and treatment. Therefore, it is a major concern for developed countries where the detection is carried out in the shortest possible time to allow for a better follow-up of infected individuals [74,75,76]. Currently, there are some near-patient tests and POCTs that allow the timely diagnosis of CT infection and are associated with better treatment [65]. In developing countries, these advanced molecular technologies are not available due to the associated costs that cannot be borne by the economies of these countries. This is a major challenge that must be overcome in order to reach all countries with the screening for this infection that allows early detection, timely treatment, a reduction in spread, the prevention of complications, and education about STIs [84].

Currently, in some countries, such as the USA, massive screening is being carried out targeting sexually active young women (<25 years) and older women if they are considered at risk, and this has produced good results, as shown by the US Preventive Services Task Force (USPSTF) study [85,86]. In addition, the USPSTF recommends retesting 3 months after treatment for positive individuals to avoid possible re-infection [86]. It is noteworthy that Wong and colleagues conclude in their systematic review that the screening must focus on young adults, regardless of their gender and risk behavior [87]. In Portugal, prevention campaigns and CT screening program are not available; thus, the majority of the population is not aware of this STI. The reason for the lack of investment in this particular problem could be the very limited data on the CT prevalence, due to its asymptomatic nature, combined with the lower testing rate and the insufficient sensitivity of some of the detection methods used (<80%) [88,89,90]. Nevertheless, many other industrialized countries, such as the UK, France, Canada, Malta, and Australia, follow the CDC guidelines for screening for chlamydia in sexually active young adults (<25 years) [91].

Therefore, the challenge may be to show the real numbers of CT infection prevalence in order to expand the screening to other developed countries. Additionally, other strategies may be explored to reduce the cost of the detection methods of CT, or to take initiatives with potential stakeholders to increase the investment in STIs and bring screening and public awareness campaigns to developing countries. These kinds of strategies would help to ensure that individuals are well-informed about the associated risks and take necessary precautions and receive an early treatment to better control the infection [84,92].

## 4. Conclusions

CT infection is a curable sexually transmitted disease that is very prevalent throughout the world, mainly because it is commonly asymptomatic. Accordingly, screening for CT infections is currently the most effective strategy for the timely control of the disease. Detection methods for *Chlamydia trachomatis* began in the 1970s with cell culture, and, in fact, the enormous health burden of CT and other STIs was the catalyst for the revolution in laboratory techniques for detecting the infection. The first developed methodology for detecting CT was the cell culture method, which had a high specificity, but low sensitivity. Later, other methods tried to replace this cell culture method, such as immune techniques, and antibody methods, in order to fill the gaps of the previous diagnostic method. Notwithstanding, the higher sensitivity was just reached by the latter, through the development of advanced molecular techniques, especially NAATs. The use of a culture for CT was once the gold standard for a diagnostic comparison. However, advancements have been sought due to the challenges in preserving organism viability during transportation and storage across various testing environments. Additionally, the tissue culture techniques for isolating CT present difficulties in standardization, requiring technical expertise, incurring high costs, and exhibiting relative insensitivity.

Currently, there are several FDA-approved NAATs for detecting CT infections, with different characteristics. However, this advanced technology comes with higher associated costs and requires a higher staff capacity and training, as well as the laboratory conditions required. Another important disadvantage of this approach is the required turnaround time. The continuous development of CT detection tests must meet the following characteristics: compact, non-invasive, accurate, and having a fast turnaround time and durable equipment. Therefore, the evolution of CT detection methods has led to POCTs, which allow for faster detection with almost all the advantages of NAATs and have enormous potential for controlling CT and other STIs. However, these tests need an upgrade to be considered the better method for CT diagnosis. Moreover, ideally, these different approaches need to reach all populations with the highest sensitivity and specificity, although, in developing countries, there is still the economic barrier that does not allow for an early detection to allow for the adequate treatment for the control or eradication of CT and other STIs.

In conclusion, we defend that addressing the complex dynamics of CT infection requires a multifaceted approach, combining medical interventions and sexual health education. Beyond the improvement of diagnostic capabilities, the adaptation to the different socioeconomic contexts, and the awareness of the populations, environmental issues must also be considered. In fact, we believe that only by considering these factors together will it be possible to effectively reduce the global burden of CT infections and the associated consequences in the distinct landscapes mentioned.

## Data Availability

Not applicable.
